# Head and neck cancer – emerging targeted therapies

**DOI:** 10.3389/fonc.2025.1640960

**Published:** 2025-09-11

**Authors:** Yasser Abouelkheer, Aarti Bhatia

**Affiliations:** ^1^ Department of Internal Medicine, Norwalk Hospital/Yale University, Norwalk, CT, United States; ^2^ Department of Internal Medicine (Oncology), Yale School of Medicine/Yale Cancer Center, New Haven, CT, United States

**Keywords:** head and neck cancer, recurrence, metastases, advanced, targeted therapies

## Abstract

Systemic therapy remains the cornerstone of treatment for recurrent and metastatic (R/M) head and neck squamous cell cancers (HNSCC). However, there is a dearth of effective treatments beyond platinum combinations, anti-programmed death-1 (PD-1) agents and the epidermal growth factor receptor (EGFR)-targeting monoclonal antibody cetuximab. Recent years have seen several exciting new agents being tested in clinical trials. These are designed to target alternate oncogenic signaling pathways and have novel mechanistic compositions, including bi-specific antibodies and antibody-drug conjugates. This review will delve into the clinical limitations of currently approved systemic therapies, explore newer agents in development and highlight ongoing clinical trials using targeted therapies in this disease.

## Introduction

Squamous cell cancers of the head and neck (HNSCC) are a heterogeneous group of malignancies that develop in the upper aerodigestive tract, which includes the oral cavity, pharynx, and larynx. This disease accounts for 4.7% of cancer-related deaths worldwide and ranks as the sixth most common malignancy (1). Risk factors for HNSCC include excessive tobacco or alcohol use and oncogenic viral infections, such as the human papillomavirus (HPV) and Epstein-Barr virus (EBV) (2). Despite a gradual decline in smoking rates, the overall incidence of HNSCC continues to rise, driven largely by HPV-associated oropharyngeal cancers (3). More than 60% of HNSCC tumors are diagnosed at a locally advanced stage and are treated with curative intent therapy. This treatment is tailored to the tumor’s extent, the primary tumor site, and the risk of functional impairment. For early-stage disease, either single-modality surgery or radiation therapy (RT) is typically sufficient, with the choice depending on functional assessment and patient preference. In contrast, aggressive multimodal treatment is used for locally advanced disease. Nevertheless, up to 40% of patients may still experience locoregional recurrences and/or distant metastases (4, 5).

Systemic therapy is the mainstay of treatment for unresectable locoregionally recurrent HNSCC as well as for distant disease. In 2006, cetuximab became the first, and to-date, only targeted therapy to be FDA approved for the treatment of HNSCC. This approval came on the basis of improved locoregional control (LRC) and overall survival (OS) when used in combination with RT versus RT alone for patients with locally advanced disease (6). It was also approved in the second-line treatment of recurrent or metastatic (R/M) HNSCC, after progression on platinum-based chemotherapy, based on a 13% overall response rate (ORR) in a multi-center phase 2 trial (7). And in 2011, cetuximab was granted frontline approval in combination with platinum-fluorouracil chemotherapy in R/M HNSCC based on improved OS compared to platinum doublet chemotherapy alone in the phase 3 EXTREME trial (8, 9).

Subsequently, agents targeting the immune checkpoint programmed death-1 (PD-1), were investigated in R/M HNSCC. Nivolumab and pembrolizumab both demonstrated clinical activity in platinum-resistant patients in the CheckMate-141 and KEYNOTE-012 trials respectively and were granted FDA approval in the second-line setting in 2016 (10, 11). KEYNOTE-048 was a randomized phase 3 trial which demonstrated improved OS in patients randomized to pembrolizumab plus chemotherapy compared with cetuximab plus chemotherapy as well as improved OS in the subgroup of patients with programmed death-ligand-1 (PD-L1) combined positive score (CPS) ≥1 HNSCC, when randomized to pembrolizumab as a single agent compared with cetuximab plus chemotherapy (12). This trial led to pembrolizumab being approved in the first-line treatment of R/M HNSCC in 2019. Despite these recent advances in systemic therapy, median OS (mOS) for patients diagnosed with R/M HNSCC is approximately 13 months, and there is a critical unmet need for more efficacious and well-tolerated agents and combinations (13).

Recent developments in HNSCC treatment have focused on testing novel combinations of immune checkpoint therapies as well as targeted therapies, with the intent to benefit a greater proportion of patients. The remainder of this review will focus on promising targeted therapies and their mechanisms of action.

## Role of epidermal growth factor receptor targeting in HNSCC

EGFR is a transmembrane glycoprotein receptor that is a member of the Erythroblastic Leukemia Viral Oncogene Homolog (ErbB) family of receptor tyrosine kinases (RTKs). Up to 90% of HNSCC cases demonstrate overexpression of EGFR, and this has been linked to treatment resistance and poor prognosis (14). The binding of the EGF and transforming growth factor-alpha (TGF-α) ligands and the subsequent activation of EGFR signaling pathways initiate a cascade of intracellular processes that promote proliferation and metastasis via the RAS/RAF/MAPK pathway, survival and therapeutic resistance through the PI3K/AKT/mTOR pathway, and immune evasion and angiogenesis through the JAK/STAT pathway (15, 16).

Given the central role of EGFR in HNSCC tumor biology, multiple agents have been tested to inhibit this signaling pathway. Most importantly, the only currently approved therapy, Cetuximab, is a chimeric monoclonal antibody (mAb) that blocks ligand binding to the extracellular domain of EGFR. The effect of Cetuximab extends beyond the inhibition of EGFR signaling. It engages immune effector cells via its Fc region, triggering the release of cytotoxic molecules and resulting in cancer cell death, a mechanism known as antibody-dependent cellular cytotoxicity (ADCC) (17–21). However, only a small minority of patients benefit from cetuximab monotherapy, and responses are not durable (7). Panitumumab has emerged as an alternative monoclonal antibody to cetuximab. While it binds to the extracellular domain of the EGFR, it has limited ADCC compared to cetuximab. The CONCERT-1 trial, which enrolled patients with locally advanced HNSCC, found no benefit with adding panitumumab to standard chemoradiation (22). Furthermore, the CONCERT-2 trial demonstrated that panitumumab was less effective than cisplatin when combined with RT (23). Therefore, panitumumab is not routinely recommended for the treatment of HNSCC. EGFR tyrosine kinase inhibitors (TKIs) such as erlotinib, gefitinib, and afatinib are small molecules that bind competitively to the ATP-binding domain in the intracellular portion of EGFR, inhibiting autophosphorylation and the subsequent downstream signaling of the receptor (24, 25). Despite promising preclinical studies, most TKIs have demonstrated limited efficacy in clinical trials in HNSCC (26).

**Figure 1:**
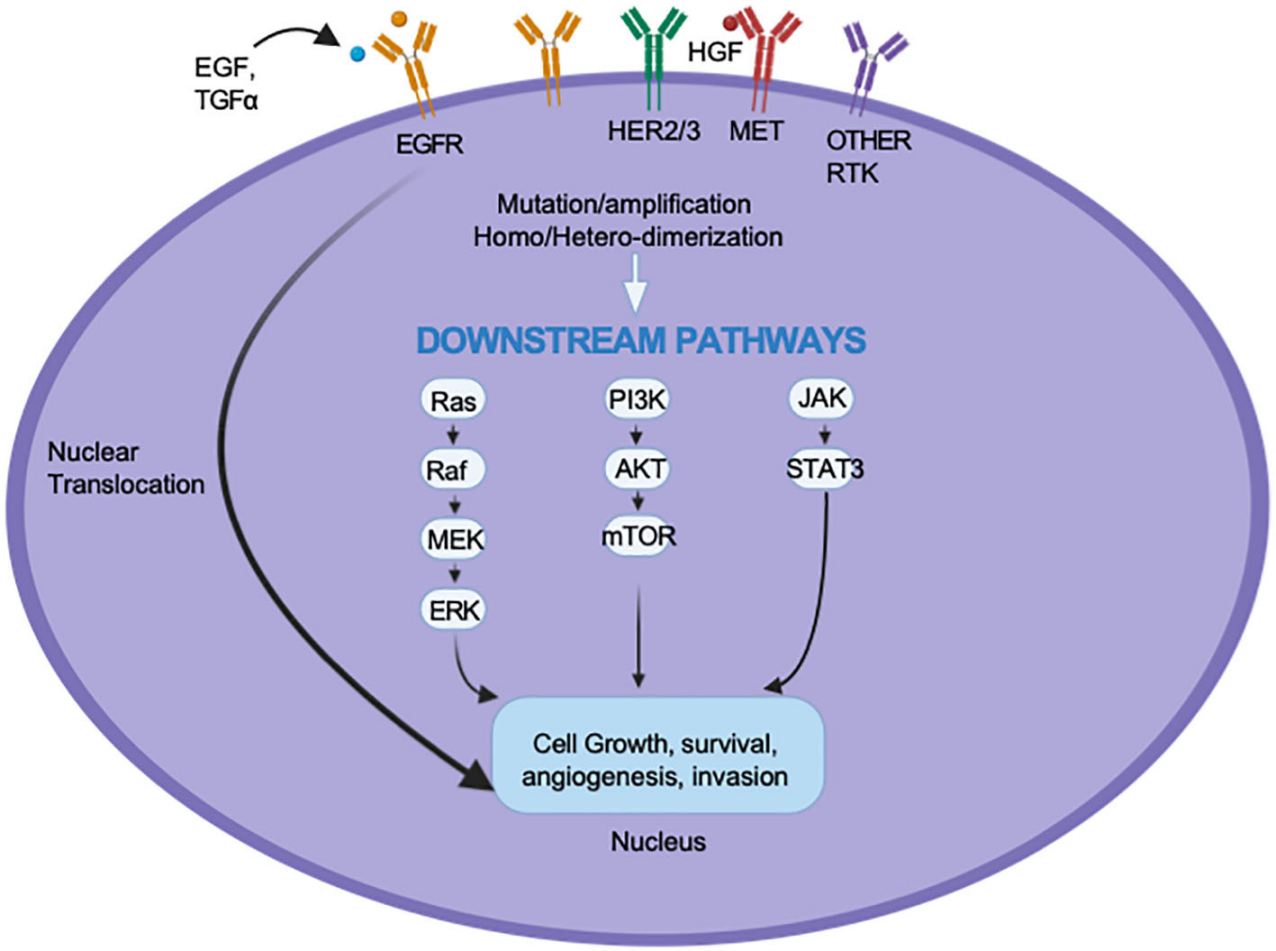
Mechanisms of resistance to EGFR inhibition.

To better understand why only a subset of patients respond to EGFR inhibition and why even responders rarely achieve a lasting effect, preclinical studies have been conducted to clarify the resistance mechanisms that hinder the effectiveness of cetuximab and TKIs. These mechanisms are categorized as inherent; those associated with the tumor’s genomic makeup, and acquired, which develop in response to therapy (see **Figure 1**). Inherent resistance mechanisms include *de novo* mutations of downstream proteins such as PI3K, KRAS, and BRAF, resulting in EGFR-independent activation of their associated pathways (27–29). Inherent resistance also exists through alternative compensatory RTK pathways such as HER2, MET, and IGF-1R (30–32). The activation of these alternative oncogenic pathways allows tumors to maintain proliferation, survival, and immune evasion despite EGFR inhibition.

**Figure 2:**
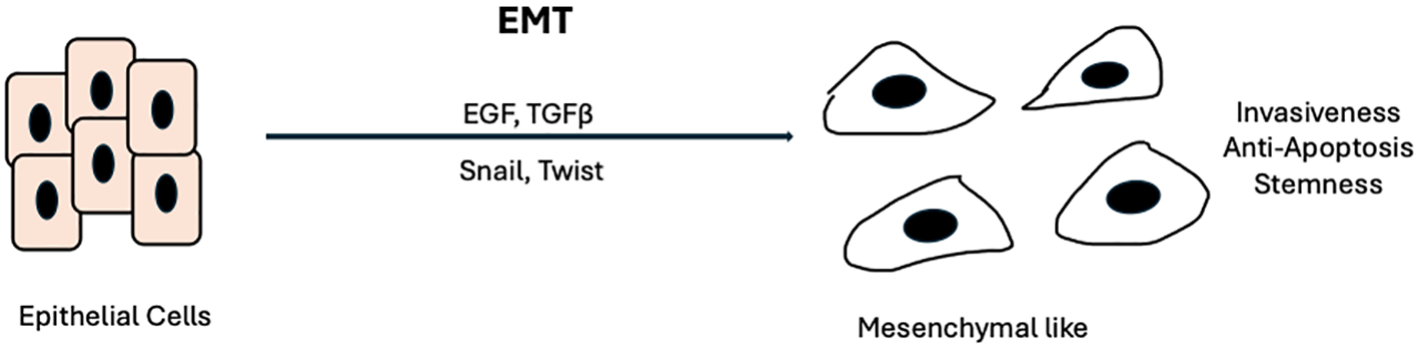
Epithelial-mesenchymal transition.

On the other hand, acquired resistance involves mechanisms that occur in response to treatment and typically reflect the evolution of tumor biology. A prime example of acquired resistance in EGFR is the well-studied gatekeeper mutation T790M in non-small-cell lung cancer (NSCLC). This mutation, found in EGFR exon 20, accounts for up to 60% of resistance to Gefitinib in NSCLC (33, 34). It increases the affinity of ATP for binding to EGFR, which impairs gefitinib’s ability to inhibit EGFR signaling. In response, the third-generation TKI Osimertinib was developed to preferentially bind and overcome the effects of mutant EGFRs, including those harboring the T790M mutation in NSCLC. Osimertinib has had great success in NSCLC, with evidence of improved progression-free survival (PFS), objective response rate (ORR), and intracranial response rate in the AURA2 and AURA3 trials (35, 36). However, in HNSCC, the genomic landscape differs from that in NSCLC. Activating mutations like those in T790M are rare in HNSCC. Rather, HNSCC cells tend to upregulate and overexpress preexisting RTK pathways, including HER2, HER3, and MET (37–39). Additionally, a key process in HNSCC resistance and metastasis is the epithelial-to-mesenchymal transition (EMT), during which neoplastic epithelial cells acquire mesenchymal properties that enable them to migrate, seed, and propagate throughout the body (40, 41). Key factors in EMT include EGF and TGF-β. The binding of these ligands to their respective receptors (EGFR and TGF-β-R) activates transcription factors such as Snail, Twist, and Slug, leading to the disruption of cell-cell adhesion, impaired apical-basal cell polarity, and upregulation of mesenchymal proteins (42–44) (**Figure 2**).

## Next-generation EGFR inhibitors

Beyond Cetuximab and tyrosine kinase inhibitors (TKIs), additional EGFR inhibitors have been developed, with Nimotuzumab being a notable example. Nimotuzumab is a novel humanized monoclonal antibody that targets EGFR (45). Like Cetuximab, Nimotuzumab binds to the extracellular domain of EGFR, preventing its ligands, EGF and TGF-α, from attaching and activating the receptor (46). However, unlike Cetuximab, which binds strongly and monovalently to individual EGFR molecules, Nimotuzumab binds bivalently and with intermediate strength (47). Nimotuzumab thus requires attachment to two EGFR molecules on the cell surface, allowing it to selectively target cells with moderate to high levels of EGFR expression. This unique binding mechanism helps minimize off-target receptor interactions, thereby reducing potential side effects. Furthermore, nimotuzumab has been shown to maintain the active conformation of the EGFR receptor, which is necessary for ligand-independent basal signaling and essential for normal cell function (45).

Early phase I and II trials with Nimotuzumab demonstrated that it is better tolerated, particularly from a standpoint of dermatologic adverse events (48). Since EGFR is highly expressed in skin epithelial cells, dermatologic toxicities are common with EGFR inhibitors, especially during the initial weeks of treatment (49, 50). However, due to its unique binding method, Nimotuzumab has demonstrated a significantly lower incidence of infusion reactions and skin-related toxicities (51). A single-center phase III randomized clinical trial comparing cisplatin-based chemoradiation (CRT) alone versus CRT with Nimotuzumab in 536 patients with newly diagnosed, treatment-naïve, locally advanced HNSCC showed improved PFS (hazard ratio (HR) 0.69; P = .004), disease-free survival (DFS) (HR, 0.71; P = .008), and a trend to improved OS (HR, 0.84; 95% CI, 0.65-1.08; P = .163) with the addition of nimotuzumab (52). A meta-analysis of randomized controlled trials, including 1012 cases of locally advanced HNSCC and comparing Nimotuzumab combined with RT or CRT to CRT alone or RT alone also showed improved OS (HR 0.75, P<0.05), PFS (HR 0.69, P<0.05), ORR (Risk Ratio [RR] 1.32, P<0.05), and complete response rate (CRR) (RR 1.52, P<0.05) with the addition of nimotuzumab (53).

While not FDA-approved for clinical use in the United States (US), Nimotuzumab is used in the treatment of HNSCC in other countries such as India, China, and Argentina.

## Approaches combining EGFR inhibitors

Recent trials have investigated the vertical inhibition of EGFR signaling through the combination of a mAb and a TKI. In a phase 2 trial involving 24 patients with treatment-naïve R/M HNSCC, the combination of chemotherapy, cetuximab, and erlotinib (added starting with cycle 2) resulted in an ORR of 58% and a median PFS (mPFS) of 5.2 months. When compared to historical data from the EXTREME trial, this dual-blockade approach achieved a relatively high response rate. Importantly, it also demonstrated a tolerable safety profile, with the most common toxicities being anemia, neutropenia, and skin rash (54). Similarly, in a single-arm phase 2 study that enrolled 50 patients, the majority of whom had platinum- and anti-PD-1-refractory R/M HNSCC, the combination of cetuximab and afatinib resulted in an ORR of 23.4%. This response was primarily driven by the p16-negative subgroup, which had an ORR of 38.5% and a mPFS of 3.8 months. In contrast, the p16-positive cohort had a mPFS of 1.8 months. The most common adverse events reported included diarrhea, anemia, and rash (55). This suggests that dual EGFR blockade with an anti-EGFR mAb and TKI could potentially overcome cetuximab resistance for some patients, particularly those with HPV-negative disease.

Another Phase 2 randomized trial compared cetuximab and afatinib in 124 patients with platinum-refractory R/M HNSCC, permitting crossover to the other treatment arm upon disease progression or intolerable adverse events. The response was assessed by both the investigator (IR) and an independent central review (ICR). The ORR was 16.1% for afatinib and 6.5% for cetuximab by IR (P = 0.09), while the rates by ICR were 8.1% for afatinib and 9.7% for cetuximab (P = 0.78). Disease control rate (DCR) was 50% for afatinib and 56.5% for cetuximab by IR (P = 0.48). After crossover, DCR was 38.9% for patients who switched from cetuximab to afatinib and 33.3% for those switching from afatinib to cetuximab by IR, while both groups showed an 18.8% control rate by ICR. This suggests a partial non-cross-resistance between the two EGFR inhibitors, potentially allowing for an extension of clinical benefit. However, drug-related adverse events (DRAEs) in 23% of patients treated with afatinib led to treatment discontinuation, indicating an unfavorable side effect profile (56).

## Combining monoclonal antibodies targeting different RTKs

Blocking EGFR with cetuximab is limited by compensatory signaling through parallel RTKs. A key strategy to overcome this limitation is to combine multiple mAbs targeting different RTKs, which helps block crosstalk and aids in resensitizing resistant tumors. Dysregulation of c-MET signaling, triggered by its ligand hepatocyte growth factor (HGF), has been implicated in driving cetuximab resistance, particularly in HPV-negative HNSCC (57). Recent trials have investigated targeting the HGF/c-MET pathway for its therapeutic potential. In a multicenter, non-comparative Phase 2 trial involving 58 patients with platinum- and cetuximab-refractory R/M HNSCC, patients were assigned to receive either ficlatuzumab (an anti-HGF IgG1) alone or in combination with cetuximab. The combination arm achieved a mPFS of 3.7 months and an ORR of 19%. Notably, the HPV-negative cohort experienced the most significant benefit, with an ORR of 38% and a mPFS of 4.1 months. This benefit was further enriched in cases with high c-MET expression. However, the monotherapy arm demonstrated futility and was therefore discontinued early. The most commonly observed adverse events in the combination group included acneiform rash, hypoalbuminemia, and edema (58). Based on these findings, a global double-blind phase 3 trial, (FIERCE-HN) is currently enrolling patients (NCT06064877). This trial compares the effectiveness of cetuximab combined with ficlatuzumab against cetuximab combined with a placebo. The results may be practice-changing and could guide future strategies for targeting parallel signaling pathways to address cetuximab resistance.

Recent studies have investigated the role of HER3 (ErbB3) as a RTK to overcome resistance to cetuximab. In a multicenter, Simon two-stage phase 2 trial involving 30 patients with HPV-negative, cetuximab-resistant R/M HNSCC, cetuximab was combined with an anti-ErbB3 monoclonal antibody CDX-3379. The ORR was 6.7% (2/30), and the mPFS was 2.2 months. Unfortunately, this combination was associated with high toxicity, as 53% of patients experienced grade 3 or higher treatment-related adverse events, leading to dose reductions in 70% of cases. Although the concept of dual targeting of EGFR and ErbB3 appeared promising from a mechanistic perspective, the clinical results showed only modest efficacy and high toxicity, making it unsuitable for further development (59).

Combinatorial strategies in RTK targeting can be effective; however, success may hinge on key factors such as selecting combinations with acceptable tolerability and targeting the right compensatory pathways in genomically preselected patients.

## Bispecific antibodies in HNSCC

With advancements in antibody engineering, novel bispecific antibodies (BsAbs) are an emerging group of drugs being investigated in the treatment of HNSCC. BsAbs can target and crosslink two distinct epitopes, either on the same cell or on two nearby cells. There are two major classes of BsAbs currently being tested in HNSCC: the first is dual-targeting BsAbs, which bind to two different antigens expressed on cancer cells. The second class is T-cell engagers (TCEs), which bind a T-cell receptor, such as CD3, and a tumor-associated antigen. This interaction stimulates targeted cytotoxicity against cancer cells (60).

In 2024, the FDA granted Petosemtamab (formerly MCLA-158) a breakthrough therapy designation. This BsAb targets both EGFR and the leucine-rich repeat-containing G-protein-coupled receptor 5 (LGR5), a stem cell marker linked to the Wnt signaling pathway. Petosemtamab was identified through large-scale functional screening in patient-derived organoids and demonstrated an ability to trigger EGFR degradation and inhibit growth in colorectal cancer cells (61). In addition, it possesses enhanced ADCC and antibody-dependent cellular phagocytosis (ADCP) activity, promoting recognition and elimination of malignant cells. In a phase 2 study of Petosemtamab monotherapy in patients with platinum- and anti-PD-1-refractory R/M HNSCC, the ORR was 40.4% (19 out of 47 patients), the mPFS was 5.1 months, and the mOS was 12.5 months. The most common treatment-emergent adverse event observed was acneiform dermatitis, occurring in 37% of patients (62). When Petosemtamab was combined with pembrolizumab as a first-line treatment for PD-L1-positive R/M HNSCC, the recently updated ORR was 60% (26 out of 43 patients). Here, the median duration of response was 11 months, and the Kaplan-Meier estimate of OS at 6 months was 93% (mOS was not reached). The most frequent adverse events were acneiform dermatitis (49%), asthenia (49%), and rash (44%) (63). While the pivotal phase 3 trial (NCT06525220) is underway, the early, promising findings for Petosemtamab position it at the forefront of its class.

A key strategy employed by the novel BsAbs is simultaneously targeting different RTK pathways. This strategy seeks to prevent compensatory upregulation of alternative RTK pathways when one pathway is inhibited, thereby preventing drug resistance. BCA101 (ficerafusp) is another promising BsAb, which is designed by fusing an anti-EGFR mAb with the extracellular binding domain of a TGF-β receptor. The anti-EGFR component of BCA101 functions similarly to cetuximab by binding to and blocking EGFR. Meanwhile, the TGF-β binding domain serves to sequester TGF-β molecules. This unique first-in-class bifunctional design allows for the inhibition of two major signaling pathways involved in HNSCC growth, survival, and immune evasion. In xenograft models, BCA101 could localize to tumors, neutralize 90% of TGF-β molecules, and show durable tumor growth suppression (64). A phase 1 trial of BCA101 alone or combined with pembrolizumab in advanced solid tumors demonstrated tolerability and safety. The most common adverse effect was rash (70%), in addition to fatigue, pruritus, and epistaxis (65). Building on these results, a dose expansion study (NCT04429542) of combination BCA101 and pembrolizumab in thirty-nine efficacy-evaluable patients with treatment-naïve, R/M HNSCC and with tumor PD-L1 CPS ≥ 1 reported an ORR of 54%. Most of the benefit was observed in HPV-negative patients; this subset had a confirmed ORR was 64%. Median PFS was 7.4 months for the entire cohort and 9.8 months in the HPV-negative subset. The median OS rate was 61.5% (66). The most common adverse event of any grade was an acneiform rash, occurring in 75% of the patients (67). The randomized phase 2/3 FORTIFI-HN01 trial is currently enrolling patients to investigate this combination in the first-line treatment of R/M HNSCC (NCT06788990).

Another agent in development, SI-B001, is a BsAb designed to target both EGFR and HER3. *In vivo* studies using xenograft models demonstrated that SI-B001, when used as a monotherapy, is more effective than cetuximab in inhibiting tumor growth. Furthermore, when SI-B001 was combined with carboplatin and paclitaxel in xenograft models, it produced a synergistic antitumor effect that surpassed the results of cetuximab used alongside the same agents (68). Two phase II clinical trials reported promising results with SI-B001 in R/M HNSCC. The S209 monotherapy trial evaluated SI-B001 alone in patients with R/M HNSCC who had progressed on prior anti-PD-1/L1 therapy plus platinum-based chemotherapy and demonstrated an ORR of 22.2% (2/9) with a mPFS of 2.7 months (95% CI: 1.8-7.9) (69). Hypomagnesaemia was the most common grade ≥3 treatment-related adverse event (TRAE) in the monotherapy trial, with an incidence of 9%, suggesting a good safety profile but modest efficacy in heavily pretreated HNSCC patients. The S206 combination therapy trial also included patients with R/M HNSCC who had progressed on prior anti-PD-1/L1 therapy, either alone or in combination with platinum-based chemotherapy, and had received ≤2 prior lines of treatment. In this trial, patients were administered either SI-B001 combined with paclitaxel (Group A) or SI-B001 combined with docetaxel (Group B). Group A had an ORR of 64.3% with a mPFS of 5.6 months (95% CI: 5.1-6.3). Group B had an ORR of 12.5% with a mPFS of 1.9 months (95% CI: 1.2-3.7). The combination of SI-B001 with paclitaxel was therefore determined to be worthy of further investigation.

Additional BsAbs are currently being developed to target various RTK pathways in HNSCC. A notable example is the targeting of the mesenchymal-epithelial transition factor c-MET, a proto-oncogene recognized for its role in promoting oncogenesis in several malignancies, including HNSCC. c-MET is overexpressed in HNSCC and is particularly relevant in HPV-negative disease, as it drives survival, proliferation, and metastasis (70). The binding of the HGF ligand to its receptor c-MET activates major signaling kinases such as MAPK and PI3K-associated pathways, activating downstream cell cycle regulators (71). MCLA-129 is a new BsAb that is currently under investigation, specifically designed to target both EGFR and c-MET. Like previously discussed BsAbs, MCLA-129 is engineered with enhanced ADCC and ADCP activity (72). In a phase 1/2 trial (NCT04868877), MCLA-129 was administered in 18 R/M HNSCC patients every two weeks in 28-day cycles (73). The median duration of exposure was 8 weeks. Among the 12 evaluable patients, 17% (2/12) achieved an unconfirmed partial response, with a disease control rate (DCR) of 67% (95% CI: 35-90%). The most common adverse events included infusion-related reactions (72%) and skin toxicity (61%).

As a class of therapeutics, BsAbs have the potential to advance the management of HNSCC. Perhaps the most promising and farthest along in development are Petosemtamab and Ficerafusp. Preliminary efficacy results of these agents in combination with pembrolizumab have demonstrated their ability to significantly improve response rates and survival, particularly in the high-risk HPV-negative population. However, the simultaneous targeting of different receptors that are ubiquitously expressed in both malignant and healthy tissues increases the risk of off-target effects and overlapping toxicities, leading to a broader range of side effect profiles. Current early-phase clinical trials lack long-term efficacy data, resulting in a limited understanding of the durability of responses to these therapies. Previous experience, for instance with the LEAP-010 trial of pembrolizumab plus Lenvatinib, has taught us that not all promising early-phase trial combinations go on to succeed in a phase 3 trial setting, possibly due to treatment-related toxicities necessitating dose reduction or treatment discontinuation (74). Thus, larger phase 2 and 3 trials with extended follow-up are essential to validate current studies’ safety and efficacy.

## Antibody-drug conjugates

Among the many exciting developments in cancer therapies, Antibody-Drug Conjugates (ADCs) are particularly promising, as they combine the therapeutic potential of targeted therapy with that of cytotoxic chemotherapy. ADCs are designed using an mAb covalently linked to a cytotoxic payload, targeting a specific tumor antigen (75). Once the ADC binds the cell surface receptor, the ADC-receptor complex is endocytosed, allowing for the delivery of the cytotoxic payload intracellularly. The payload can also permeate the cell membrane and exert its anti-cancer effect on surrounding bystander cells (76). In the past decade, ADCs have shown clinical efficacy against various solid tumors, including breast, cervical, gastric, urothelial, and ovarian cancers. They have also proven effective in treating hematological malignancies, such as acute myeloid leukemia, hairy cell leukemia, diffuse large B-cell lymphoma, and Hodgkin lymphoma. Many of these treatments have received FDA approval (77–79).

Currently, there are no approved ADC therapies for clinical use in HNSCC, but several agents are undergoing investigation in phase 1 and 2 trials. One such agent is MRG003 or becotatug vedotin, a humanized anti-EGFR IgG1 that is conjugated to monomethyl auristatin E (MMAE) via a cleavable valine-citrulline linker. In a phase 2a trial involving patients with R/M nasopharyngeal carcinoma who had previously failed platinum-based and/or PD-L1 therapies, MRG003 demonstrated an ORR of up to 55.2% and a DCR of 86.2% at the higher dosage of 2.3 mg/kg. The most frequently reported TRAEs were dermatological, with 49.2% of patients experiencing a rash (80). In a Phase 1/2 study that combined MRG003 with Pucotenlimab, a recombinant humanized PD-1 inhibitor, antitumor effects were observed. In treatment-naïve patients with EGFR-positive HNSCC, the combination regimen achieved an ORR of 60% and a DCR of 80%. In a recently reported randomized trial of 173 R/M, heavily pre-treated nasopharynx cancer (NPC) patients received MRG003 2.3 mg/kg every 3 weeks or investigator’s choice chemotherapy (81). ORR was 30.2% in the MRG003 arm versus 11.5% with standard chemotherapy (p value 0.0025), median PFS was 5.82 months versus 2.83 months with chemotherapy (p value 0.0146) and OS data was not mature. Collectively, this data suggests its potential to provide significant antitumor activity in both treatment-naïve and heavily pretreated HNSCC and NPC patients, and more so when administered in combination with an anti-PD-1 agent.

Another agent in early investigation is ozuriftamab vedotin (BA3021), a conditionally binding ROR2-ADC, using MMAE as the cytotoxic payload (82). ROR2 is a transmembrane protein RTK enriched in several tumor types. In HNSCC, its overexpression is driven by HPV-associated E6 and E7 oncoproteins (83). Ozuriftamab vedotin is an ADC designed to bind to ROR2 under low pH conditions of the tumor microenvironment, thus reducing off-target toxicity by sparing normal tissue and improving pharmacokinetics (84). In a phase 1 trial (NCT03504488), the recommended phase 2 dose was established at 1.8 mg/kg. This dose was tested in two different schedules in a phase 2 trial in 40 patients with R/M, chemotherapy- and anti-PD-1 refractory HNSCC. Every two weeks dosing was found to be tolerable and effective. Among 11 evaluable patients with HPV-associated HNSCC, ORR was 45%, median PFS was 4.8 months and median OS was 11.6 months. Most adverse events were low grade, commonest high-grade events were nausea, diarrhea, cytopenias and neuropathy.

Other emerging ADC therapies include tisotumab vedotin and enfortumab vedotin. Tissue factor is known to be aberrantly expressed in various squamous tumor cells, including HNSCC. Tisotumab vedotin (TV) is a first-in-class ADC that was developed by linking an anti-tissue factor IgG1 antibody with the antimitotic payload MMAE. Once the ADC is internalized by tumor cells, it triggers apoptotic cell death and induces bystander cytotoxicity (85). In the phase 2 InnovaTV 207 trial, TV treatment in 40 patients with R/M HNSCC demonstrated an ORR of 32.5%. The median time to response was 1.4 months, and the DOR was 5.6 months. Grade three or higher TRAE were observed in 25% of patients, with peripheral neuropathy being the most common, affecting 12.5% of patients. These findings suggest a clinically meaningful and durable response in pan-refractory R/M HNSCC with a tolerable safety profile for TV (86).

Nectin-4 is expressed in up to 86.2% of HNSCC and is significantly enriched in p16-positive tumors and never-smokers (87). The ADC enfortumab vedotin (EV) is an anti-Nectin-4 IgG1 antibody conjugated to MMAE. In the single-arm, two-stage Phase 2 EV-202 trial, 46 patients with R/M HNSCC received treatment with EV and were followed for a median duration of 9.3 months. The ORR was 23.9%, with a DCR of 56.5% and a mPFS of 3.9 months. Common TRAEs included alopecia, fatigue, and peripheral neuropathy. Notably, 34.8% of patients experienced Grade three or higher TRAEs, which included anemia and neutropenia. This data justifies its further evaluation in phase 3 trials. Additional studies exploring combination strategies, including EV or TV with checkpoint blockade, may uncover further potential of these therapies in HNSCC.

Lastly, Sacituzumab govitecan is an ADC targeting trophoblast cell-surface antigen 2 (Trop-2) that was investigated in the phase 2 TROPiCS-03 basket trial in patients with treatment-refractory HNSCC (88). The primary endpoint was investigator-assessed ORR. Forty-three patients were treated and the ORR for the cohort was 16%. Commonest treatment-emergent adverse events were diarrhea, nausea and neutropenia.

## Other targeted therapies in HNSCC

Phosphatidylinositol 3-kinase (PI3K)-mTOR signaling pathway activation is a known mediator of treatment resistance and disease progression in HNSCC (89). It can drive primary or secondary resistance to paclitaxel by increase in protein kinase B (AKT) activity (90). Buparlisib is an oral pan-PI3K inhibitor and in HNSCC xenograft models, led to down-regulation of PI3K–mTOR pathway signaling, with reduced tumor hypoxia and vascular remodeling (91). The combination of buparlisib and paclitaxel showed promising signs of clinical activity in a phase 1B trial in advanced solid tumors (92). Subsequently, a randomized, blinded study, BERIL-1 was conducted in patients with platinum-pretreated R/M HNSCC (93). 158 patients were enrolled and randomized to receive paclitaxel with either buparlisib or placebo. Median PFS was 4.6 months in the buparlisib group versus 3.5 months in the placebo group (HR 0.65, p = 0.011). commonest grade 3–4 adverse events were hyperglycemia, cytopenias and fatigue. Based on these findings, the confirmatory phase 3 BURAN trial enrolled 487 patients with R/M HNSCC who have progressed on anti-PD-(L)1-based treatment (94). Primary endpoint was OS, and the company recently announced that the study failed to meet its endpoint compared to paclitaxel alone (95).

Dysregulated activation of the cyclin-dependent kinase 4 and 6 (CDK4/6) and cyclin D1 regulatory complex is known to drive the cell cycle and tumor progression, especially in HPV-unrelated HNSCC. CDK4/6 hyperactivation also mediates cetuximab resistance. In preclinical models of HPV-negative HNSCC, CDK4/6 inhibition decreased tumor growth and in combination with cetuximab, synergistically reduced viability of cell lines (96). Phase 1 and 2 trials established the safety of co-administering the selective CDK4/6 inhibitor palbociclib and cetuximab in patients with R/M HNSCC and showed an ORR of 19% in cetuximab-resistant patients and 39% in platinum-resistant patients (97, 98). A phase 3 trial (NCT04966481) is currently underway evaluating this combination in CDKN2A-altered, HPV-unrelated HNSCC (99).

Other recent trials have explored genotype-directed therapies for patients with HNSCC. HRAS mutations are particularly enriched in HPV-negative HNSCC and are associated with poor clinical outcomes (100). Tipifarnib is a new oral medication that acts as a highly selective farnesyl-transferase inhibitor. It prevents the farnesylation of HRAS and its anchoring to the cell membrane, thereby inhibiting MAPK signaling and promoting tumor apoptosis (101). In the signal-seeking RUN-HN phase 2 study (NCT02383927) involving patients with R/M HNSCC with high variant allele frequency (VAF) mutated HRAS, ORR was 55%, with a mOS of 15.4 months. These results led to the pivotal AIM-HN trial (NCT03719690), which showed an ORR of 30% based on investigator assessment and 20% based on independent review, along with a mPFS of 2.6 months (independent review) (102–104). Although additional data is still pending, tipifarnib presents a potential biomarker-driven oral therapy for a subset of R/M HNSCC patients with HRAS mutations.

## Early phase trials of targeted therapies

There has been a notable increase in early-phase clinical trials in HNSCC over the past decade. This surge is driven by the discovery of new molecular targets and the introduction of innovative therapy classes, such as BsAbs and ADCs. One example is amivantamab (JNJ-61186372), a BsAb designed to engage EGFR and c-MET (105). Amivantamab is mechanistically distinct from MCLA-129 in its binding epitope and ability to induce trogocytosis (106–108). In an ongoing multicenter phase 1/2 trial (NCT06385080), amivantamab is currently being studied alone or in combination with other treatment agents in R/M HNSCC. Anticipated adverse events of amivantamab include infusion-related reactions and the development of rash, based on NSCLC cohorts (109).

Another promising agent is the ADC, patritumab deruxtecan (U3-1402), which is developed using an anti-HER3 IgG1 conjugated to the topoisomerase I inhibitor deruxtecan (DXd) (110). Patritumab deruxtecan has a dual action: it downregulates HER3 signaling and induces DNA double-strand breaks, leading to apoptosis. By targeting HER3, this agent addresses a key escape mechanism observed with EGFR-directed therapies. The HERTHENA-PanTumor01 trial (NCT06172478) is an ongoing open-label, global phase 2 study designed to evaluate the efficacy and safety of patritumab deruxtecan in patients with R/M solid tumors, including HNSCC, excluding nasopharyngeal cancer (111). **Table 1** lists the key ongoing trials using targeted therapies in HNSCC.

**Table 1:** Summary of recent trials testing novel targeted therapies in HNSCC.

Target	Agent(s) and design	Population	Endpoints	Outcomes	Development stage
HGF/c-MET	Randomized phase 2: Ficlatuzumab (anti-HGF mAb) +/- Cetuximab	R/M HNSCC refractory to anti-PD-1 therapy and platinum-based chemotherapy	Median PFS	ORR 38% and mPFS 4.1 months in HPV-	Phase 3 FIERCE-HN trial (NCT06064877) ongoing
	Phase 1/2 trial of MCLA-129 (BsAb targeting EGFR and c-MET)	R/M HNSCC refractory to standard therapies	ORR	PR 17%, DCR 67%	Not announced
	Phase 1b/2 trial of Amivantamab (BsAb targeting EGFR and MET)	R/M HNSCC refractory to standard therapies	Not reported	Not reported	Not announced
HER3	Phase 2 trial of CDX-3379 (anti-HER3 mAb) and Cetuximab	R/M, HPV-, Cetuximab-resistant HNSCC	ORR	ORR 6.7%, mPFS 2.2 months	Development discontinued
	Phase 2 trial of SI-B001 (EGFR×HER3 BsAb) alone or in combination with paclitaxel or docetaxel	R/M HNSCC progressed on anti-PD-1 and platinum-based chemotherapy	ORR	ORR 22.2% with monotherapy, 64.3% in combination with paclitaxel and 12.2% with docetaxel	Not announced
	Phase 2 trial of patritumab deruxtecan (anti-HER3 ADC)	R/M HNSCC progressed on anti-PD-1 and platinum-based chemotherapy	ORR	Not reported	Not announced
EGFRxLGR5	Phase 2 trial of petosemtamab (EGFRxLGR5 BsAb) as monotherapy or in combination with pembrolizumab	R/M HNSCC, progressed on anti-PD-1 and platimun-based chemotherapy for monotherapy and 1L R/M HNSCC, PD-L1 CPS ≥ 1 for combination	ORR	ORR 40.4% for 2L+ monotherapy and 63% for 1L in combination with pembrolizumab	Phase 3 trials ongoing: LiGeR-HN1: randomized study of pembrolizumab versus pembrolizumab + petosemtamab in 1L R/M HNSCC (NCT06525220) LiGeR-HN2: randomized trial of petosemtamab versus investigator’s choice systemic therapy in 2L+ R/M HNSCC (NCT06496178)
EGFRxTGF- βR	Phase 1b trial of ficerafusp (EGFRxTGF- βR BsAb) in combination with pembrolizumab	R/M 1L HNSCC, PD-L1 CPS ≥ 1	ORR	ORR 54% overall and 64% in HPV-	Phase 2/3 trial ongoing: FORTIFI-HN01 in 1L, PD-L1+, HPV- R/M HNSCC (NCT06788990)
EGFR	Phase 1/2 trial of MRG003 (becotatug vedotin, EGFR targeting ADC) in combination with pucotenlimab (anti-PD-1 agent) Randomized trial of MRG003 versus investigator choice chemotherapy	Combination in 1L R/M HNSCC Randomized trial in 3L+ R/M NPC	ORR for combination trial ORR and PFS in randomized trial.	ORR 60% with combination in 1L R/M HNSCC ORR 30.2% versus 11.5% in randomized trial (p=0.0025) PFS 5.8 versus 2.8 months (p=0.0146)	Randomized phase 3 trial of MRG003 in combination with Pucotenlimab in R/M NPC (NCT06976190) planned Randomized phase 3 trial of MRG003 versus Cetuximab/​Methotrexate in patients with R/M HNSCC previously progressed on anti-PD-1 and platinum-based chemotherapy planned (NCT05751512)
ROR2	Phase 2 trial of ozuriftamab vedotin (ROR2-ADC)	R/M HNSCC progressed on anti-PD-1 and platinum-based chemotherapy	ORR	ORR 45% in HPV+ patients	Phase trial in 2L+ HPV+ R/M HNSCC planned
Tissue Factor (TF)	Phase 2 trial of tisotumab vedotin (anti-TF ADC)	R/M HNSCC previously progressed on anti-PD-1 and platinum-based chemotherapy	ORR	ORR 32.5%	Not announced
Nectin-4	Phase 2 trial of enfortumab vedotin (anti Nectin-4 ADC)	R/M HNSCC previously progressed on anti-PD-1 and platinum-based chemotherapy	ORR	ORR 23.9%	Not announced
Trop-2	Phase 2 trial of sacituzumab govitecan (anti-Trop-2 ADC)	R/M HNSCC previously progressed on anti-PD-1 and platinum-based chemotherapy	ORR	ORR 16%	Not announced
PI3K pathway	Randomized phase 3 trial of buparlisib (oral PI3K inhibitor) in combination with paclitaxel versus paclitaxel alone	R/M HNSCC previously progressed on anti-PD-1 and platinum-based chemotherapy	OS	Trial did not meet primary endpoint per press release	Negative study in HNSCC
CDK4/6	Phase 2 trial of palbociclib (CDK4/6 inhibitor) and cetuximab	R/M, HPV- HNSCC – platinum-resistant and cetuximab-resistant cohorts	ORR	ORR 39% in platinum-resistant and 19% in cetuximab-resistant patients	Randomized phase 3 trial of palbociclib and cetuximab versus cetuximab monotherapy in CDKN2A-altered, HPV- HNSCC following progression on anti-PD-1

## Discussion

The treatment landscape of HNSCC is rapidly evolving as research continues to investigate new targets and pathways. Over the last decade, pan-EGFR targeting has shown limited success in improving clinical outcomes, as reflected in the poor survival rates of patients with R/M disease. Immune checkpoint therapies have improved outcomes for some patients. However, the majority fail to derive clinical benefit and there is a critical unmet need for effective and well tolerated novel agents and combinations. Genomic profiling has identified potential targets, including tumors that are HRAS-mutant, HER3-high, and MET-co-activated. These discoveries present opportunities to address these escape mechanisms associated with EGFR therapies. However, the cumulative side effects and treatment complications associated with combination regimens remain a significant concern, especially in a heavily pre-treated patient population that may already be experiencing residual side effects from previous lines of therapies.

The lack of tumor biomarkers to identify patients who would benefit the most from targeted therapies remains a significant unmet need in HNSCC research. Decades of clinical research has taught us that the “one-size-fits-all” approach to treatment is ineffective in improving outcomes for patients. HNSCC tumors are characteristically very heterogenous, whether by location, etiology (HPV-related versus smoking-related), or biomarkers (PD-L1 expressing versus not) and we have learned that these patient and tumor traits can predict the varied biological responses to therapies. Trials are therefore increasingly being specifically designed based on tumor stage, biomarkers and prior lines of therapy. Genomic analyses of high-responding patients may offer additional predictive biomarkers for future studies. Similarly, upcoming trials could incorporate basket trial designs and stratify patients into subgroups based on molecular markers and clinical features to gain deeper insights.

Finally, despite the progress made with novel cancer therapeutics, disparities in access to these agents is a key concern, especially in low and middle-income countries (LMICs), which have a higher burden of HNSCC. Multiple recent publications have highlighted the delay in launching these products in developing countries, the dearth of real-world efficacy data in the local populations, lack of generalized medical insurance coverage and the prohibitively high out-of-pocket cost of newer drugs, as reasons why few patients are able to start and stay on treatment (112–116). Thus, as the number of clinical trials and innovative therapies increases, it is important to simultaneously think of creative solutions to bridge this affordability gap in developing countries. Some possible solutions that have been suggested include designing trials that enroll populations in LMICs, patient access and loan programs and a multi-stakeholder approach to making novel agents available in international markets.

## Conclusion

Head and neck oncology is experiencing the introduction of a wide array of new therapies, including RTK inhibitors, BsAbs, and innovative ADCs. We expect that the treatment paradigm will gradually shift from single agents targeting EGFR to genomically informed combination regimens designed to address tumor-specific escape mechanisms. Early-phase trials have demonstrated potential in re-sensitizing resistant tumors; however, establishing a durable benefit in larger confirmatory trials remains essential.
